# First-Principles Study on the Electrical and Thermal Conductivities of Cu–Zn Binary Alloys

**DOI:** 10.3390/ma18102310

**Published:** 2025-05-15

**Authors:** Lei Huang, Bo Peng, Qinchi Yue, Guojie Huang, Changhao Wang, Ruzhi Wang, Ning Tian

**Affiliations:** 1 High-Performance Copper Alloy Materials R&D Division, China Nonferrous Metals Innovation Institute (Tianjin) Co., Ltd., Tianjin 300393, China; huanglei.my@foxmail.com (L.H.); tianningnene@163.com (N.T.); 2 State Key Laboratory of Materials Low-Carbon Recycling, College of Material Science and Engineering, Beijing University of Technology, Beijing 100124, China; pengbo@emails.bjut.edu.cn (B.P.); yueqinchi@emails.bjut.edu.cn (Q.Y.); wrz@bjut.edu.cn (R.W.)

**Keywords:** Cu–Zn alloy, first-principles calculations, electrical conductivity, thermal conductivity

## Abstract

Cu–Zn alloys are widely used engineering materials with well-known industrial applications. However, studies on their electrical and thermal conductivities have primarily relied on experimental measurements, while theoretical investigations remain limited. In this work, eight crystal structure models were constructed to represent three phase configurations (α single phase, α + β′ dual phase, and β′ single phase) of Cu–Zn alloys with Zn concentrations ranging from 0 to 50 at.%. Based on the first-principles calculations combined with the Boltzmann transport equation, the electrical and thermal conductivities of these models were computed, and the electronic structure of the α-phase configurations was further analyzed. The results show that both electrical and thermal conductivities exhibit a non-monotonic trend with increasing Zn content, initially decreasing and then increasing. This trend is in strong agreement with available experimental data. Further analysis of the electronic structure reveals that, in the α-phase region, the density of states near the Fermi level is mainly contributed by Cu d-orbitals. As Zn content increases, the effective DOS near the Fermi level decreases, leading to reduced electron transport capability. For thermal conductivity, both the Wiedemann–Franz law and the first-principles calculations were employed, yielding results consistent with experimental trends. In summary, this study systematically investigates the variation of electrical and thermal conductivities of Cu–Zn binary alloys with Zn content and explores the underlying physical mechanisms from the perspective of electronic structure. The findings provide valuable theoretical support for understanding and optimizing the transport properties of complex alloy systems.

## 1. Introduction

Copper–zinc (Cu–Zn) alloys are widely used in modern industry due to their high electrical and thermal conductivity, excellent corrosion resistance, and good workability. These properties make them attractive for applications in electronics, automotive manufacturing, and piping systems. In the electronics industry, Cu–Zn alloys are commonly used in connectors and terminals for their high conductivity and thermal stability. In the field of automobile manufacturing, their superior thermal conductivity and corrosion resistance make them suitable for radiator cores, connectors, and related components [1,2,3].

The Cu–Zn alloy system exhibits complex phase transformation behavior depending on zinc content and temperature. The maximum solid solubility of Zn in Cu is approximately 38.95 wt.%. When the Zn content is below this limit, the alloy primarily shows a face-centered cubic (FCC) α-phase. As the Zn content exceeds 40 wt.%, the alloy enters the α + β two-phase region [4]. In this range, the β-phase exists as a disordered body-centered cubic (BCC) structure above 454 °C and transforms into an ordered β′-phase upon cooling below this temperature. The β′-phase retains the BCC lattice but exhibits long-range order, such as DO3 or B2-type structures [5]. With further increase in Zn content to around 50 wt.%, the β-phase becomes dominant, forming a single-phase β structure [6].

Beyond 50 wt.% Zn, additional complex intermetallic phases such as γ, δ, and ε emerge, characterized by more intricate crystal structures, significant atomic disorder, and vacancy-induced lattice relaxations. These features fundamentally change the nature of the phase transformation behavior compared to the Cu-rich side. These phase transformations significantly influence the electrical, thermal, and mechanical properties of Cu–Zn alloys [7,8,9,10,11]. From an industrial perspective, the compositional range of 0–50 wt.% Zn covers the most commercially important brasses. According to international standards such as ASTM B36 [12], widely used wrought and cast brass grades (C21000–C28000) typically contain 37–41 wt.% Zn. Alloys with Zn content above 50 wt.% suffer from severe deterioration in workability and electrical conductivity, making them unsuitable for widespread industrial applications. By adjusting the phase structure and composition, the physical properties of the alloys can be tailored, which is valuable for applications in electronics, electrical engineering, and structural components.

With the development of computational materials science, the first-principles calculations based on the density functional theory (DFT) have become widely used in metallic materials research. These methods enable the investigation of materials at the electronic structure level and have provided important insights into phase stability, electronic properties, and transformation mechanisms.

For the Cu-rich side (Zn < 50 wt.%) of the Cu–Zn system, first-principles calculations have successfully been applied to analyze order–disorder transitions between the α and β phases under various compositions and temperatures. The phase stability and transformation mechanisms have been explained from an electronic structure perspective, offering a clear understanding of their stability and evolution with composition and temperature [13,14,15,16,17,18]. However, modeling the Zn-rich side (Zn > 50 wt.%) is considerably more challenging. The presence of γ, δ, and ε phases introduces highly complex, vacancy-mediated structures and significant lattice distortions, making it difficult to construct accurate atomic models and maintain consistent evaluation criteria across different phases [11]. Moreover, the underlying physical mechanisms governing phase transformations in the Zn-rich region differ substantially from those in the Cu-rich region, further complicating theoretical investigations. Similar phase-change behavior upon crossing 50 at.% composition has also been observed in other alloy systems, such as in Sn–Sb and Cu–Ni–Cr-based materials, where doping-induced structural transitions lead to significant changes in physical properties [19,20]. Considering these factors, this study focuses on Cu–Zn alloys with Zn contents up to 50 wt.%.

Experimental data have also revealed that the electrical and thermal conductivity of Cu–Zn alloys varies with Zn content [8,9]. Specifically, both properties tend to decrease initially and then increase as Zn content rises from 0 to 50 wt.%. However, most of these findings rely on experimental methods. The lack of theoretical modeling limits understanding of the underlying mechanisms. Moreover, experimental approaches often require substantial effort in sample preparation and testing and may introduce inconsistencies due to varying conditions.

In this context, atomic models of Cu–Zn alloys in the α, α + β′, and β′ phase regions were constructed. The electrical and thermal transport properties, along with the electronic structures, are investigated using DFT combined with the Boltzmann transport equation. The influence of phase structure and composition on transport properties is analyzed in detail. This work aims to uncover the mechanisms by which different phase structures affect electron transport behavior. By correlating theoretical calculations with available experimental data, the study provides a deeper understanding of the relationship between electronic structure and transport performance.

Furthermore, recent studies on the band topological characteristics of alloy materials have confirmed the nontrivial topological band structure of the Sn–Sb alloy system. Its topologically protected surface state can significantly increase the carrier mobility and generate abnormal transport behaviors [21,22]. Although the current research on Cu–Zn alloys mainly focuses on the influence of traditional phase transitions and electronic structures on transport performance, topological band engineering provides a new idea for regulating their electrical/thermal conductivity. For instance, inducing topological quantum phase transitions through composition design or generating topological surface states by utilizing interface effects may break through the performance limits of traditional alloys [23]. The electronic structure-transport property correlation framework established in this study not only lays a foundational basis for exploring potential topological effects in Cu–Zn systems but also provides theoretical guidance for alloy design and multifunctional optimization.

## 2. Computational Methods

### 2.1. Computational Models

According to the Cu–Zn binary phase diagram [24], within the composition range investigated in this study (Zn ≤ 50 at.%), the Cu–Zn alloy system exhibits three typical phase structures with increasing Zn content: single-phase α (FCC), dual-phase α + β′, and single-phase β′ (BCC). To investigate the effects of different phases and compositions on the electrical and thermal conductivities of Cu–Zn alloys, the crystal structure models were constructed for Zn concentrations ranging from 0 to 50 at.%.

**Figure 1:**
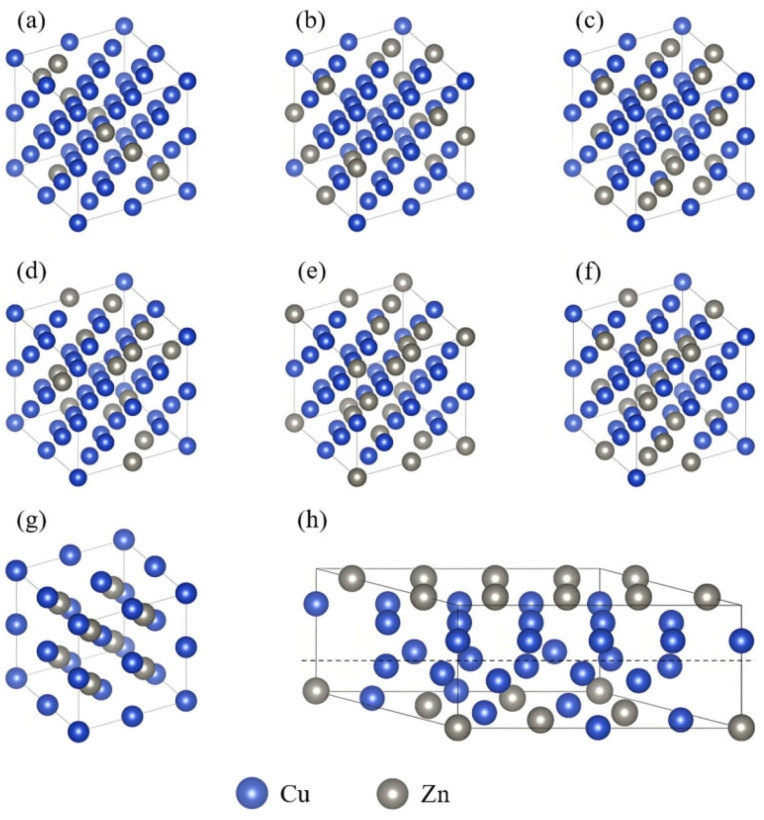
Crystal structure models of Cu–Zn alloys with different Zn content: (**a**) α phase with 12.5% Zn content; (**b**) α phase with 18.75% Zn content; (**c**) α phase with 21.875% Zn content; (**d**) α phase with 25% Zn content; (**e**) α phase with 28.125% Zn content; (**f**) α phase with 31.25% Zn content; (**g**) β′ phase with 50% Zn content; (**h**) α + β′ diphase configuration.

In the α phase, Zn atoms randomly substitute Cu atoms in a disordered solid solution with a face-centered cubic (FCC) structure. To accurately represent the atomic disorder, the alloy theoretic automated toolkit (ATAT) [25,26] was used to generate special quasi-random structures (SQS). Six α-phase structures with different Zn concentrations (12.5, 18.75, 21.875, 25, 28.125, and 31.25 at.%) were constructed, as shown in Figure 1a–f. For each concentration, 20 α-phase SQS models were generated, and their ground-state energies were calculated. Unstable structures were eliminated, and the remaining structures were subjected to molecular dynamics simulations for diffusion-annealing treatment. The most stable configuration was selected as the representative structure for each composition. The β′ phase, a Cu–Zn intermetallic compound with a body-centered cubic (BCC) structure, has a fixed Cu:Zn atomic ratio of 1:1 and exhibits an ordered solid solution, as shown in Figure 1g. The α + β′ dual-phase structure was constructed by combining the α and β′ unit cells at a defined interface. Due to the significant lattice mismatch between the α phase (a = 3.677 Å) and the β′ phase (a = 2.921 Å), the vasp.6.3.0 software was used to reconstruct the lattice vectors. The final α + β′ interface model has an interfacial spacing of 1.8 Å and an interfacial angle γ = 45°, as shown in Figure 1h. All electronic structure and transport property calculations for the Cu–Zn alloys in this study were performed based on the above models.

### 2.2. Electrical Conductivity Calculations

The electronic transport coefficients were calculated by solving the Boltzmann transport equation (BTE) under the relaxation time approximation (RTA) [27], as implemented in the BoltzTraP2 code [28]. The transport coefficients, including the electrical conductivity (*σ*) and the electronic thermal conductivity (*κ_e_*), are defined as follows:

(1) $$\sigma \left(T,\mu \right)=-\frac{\tau }{\Omega }\int_{-\infty }^{+\infty }\sigma \left(\varepsilon \right)\frac{\partial f\left(\varepsilon ,\mu ,T\right)}{\partial \varepsilon }d\varepsilon ,$$

(2) $$\kappa \left(T;\mu \right)=-\frac{\tau }{q^{2}T\Omega }\int_{-\infty }^{+\infty }\sigma (\varepsilon ){\left(\varepsilon -\mu \right)}^{2}\frac{\partial f(\varepsilon ,\mu ,T)}{\partial \varepsilon }d\varepsilon ,$$

where *τ* is the relaxation time, Ω is the unit cell volume, and *f* is the Fermi–Dirac distribution function, which is defined as follows:

(3) $$f\left(\varepsilon \right)=\frac{1}{e^{\frac{\varepsilon -\mu }{kT}}+1},$$

with *ε* being the electron energy, *μ* the chemical potential, *k* the Boltzmann constant, and *T* the absolute temperature. The transport distribution function (TDF) *σ*(*ε*) is expressed as follows:

(4) $$\sigma \left(\varepsilon \right)=\frac{1}{N}\sum_{k}\sigma_{\alpha \beta }\left(i,k\right)\delta \left(\varepsilon -\varepsilon_{k}\right),$$

where *N* is the number of *k*-points and *σ_ik_* is the conductivity tensor at each k-point [27,29], calculated as follows:

(5) $$\sigma_{\alpha \beta }\left(i,k\right)=e^{2}\tau_{i,k}v_{\alpha }\left(i,k\right)v_{\beta }\left(i,k\right),$$

where e is the elementary charge, and *v_a_* and *v_β_* are the group velocities along the *α* and *β* directions, given by (1/ℏ) (∂ϵ/∂k). BoltzTraP2 uses Fourier interpolation of the band structure obtained from VASP calculations to generate denser k-point meshes, enabling more accurate and efficient evaluation of the transport properties [30].

### 2.3. Thermal Conductivity Calculations

The thermal conductivity (*κ*) is a key parameter characterizing the heat transport capability of materials and includes contributions from both electronic (*κ_e_*) and lattice (phonon) thermal conductivities (*κ_ph_*) [31], calculated as follows:

(6) $$\kappa =\kappa_{e}+\kappa_{ph},$$

For metals where heat transport is predominantly electronic, *κ_e_* can be estimated from the electrical conductivity using the Wiedemann–Franz law [32], calculated as follows:

(7) $$\kappa_{e}=L\sigma T,$$

where *L* is the Lorenz number (typically approximated as $2.44\times 10^{-8}W\Omega K^{-2}$), *σ* is the electrical conductivity, and *T* is the absolute temperature.

### 2.4. Computational Parameters

All calculations were performed using the Vienna Ab initio Simulation Package (vasp.6.3.0) [33,34]. The projector augmented wave (PAW) method [35,36] was employed, and the exchange-correlation interactions were described using the Perdew–Burke–Ernzerhof (PBE) functional. A plane-wave energy cutoff of 400 eV was used. The total energy convergence criterion was set to 1.0 × 10^−6^ eV/atom.

k-point sampling in the Brillouin zone was performed using the Monkhorst–Pack scheme centered at the Γ point. For structure optimization, the k-point meshes were 4 × 4 × 4 for the α phase, 12 × 12 × 12 for the β′ phase, and 4 × 3 × 5 for the α + β′ dual-phase models. Geometry optimizations were carried out using the conjugate gradient algorithm [37], and the convergence criterion for atomic forces was 0.02 eV/Å.

All total energy and electronic structure calculations were based on the optimized structures. The k-point meshes used for density of states (DOS) calculations were the same as the geometry optimizations.

## 3. Results and Discussion

### 3.1. Structural Optimization Results

To verify the reliability and accuracy of our calculations, the optimized lattice parameters of pure Cu (FCC), Zn (HCP), and the Cu–Zn alloy α-phase (FCC) and β′-phase (BCC) were compared with experimental values, as shown in Table 1. The results indicate that the optimized lattice constants in this study are in good agreement with the experimental data reported in the literature [38,39,40,41,42]. The deviations in lattice parameters are within 1%, demonstrating the reasonableness and credibility of our structural optimization results.

### 3.2. Electrical Conductivity: Results and Discussion

As an important industrial alloy, Cu–Zn has been extensively studied, and considerable experimental data on its properties have been accumulated. In particular, Aoyama, S., and Ito, T., conducted a series of electrical conductivity measurements on Cu–Zn alloys in the 1940s [8], which are used here as reference standards for validating our computational results.

The nonequilibrium behavior of conduction electrons in metals can be described by the Boltzmann transport equation (BTE), which relates the response of electrons to external forces with changes in their distribution function. Due to the complexity of electron scattering processes, the relaxation time approximation (RTA) is often employed to simplify the BTE by introducing an average timescale for scattering. This approximation assumes that electrons reach a quasi-equilibrium state between scattering events, allowing their transport behavior to be described using group velocity and relaxation time.

In this work, electrical conductivity is calculated by combining the Fermi-Dirac distribution with the BTE under the RTA framework. The resulting conductivity depends on both the group velocity and the relaxation time. The group velocity characterizes the electron propagation at a given wavevector and is calculated as the gradient of the band energy with respect to the wavevector. The relaxation time reflects the dynamics of electron scattering. In this study, a constant relaxation time τ = 2.375 × 10^−14^ s is adopted based on the average values reported for Cu–Zn alloys in the literature [43]. The group velocity is obtained from the electronic band structures computed by VASP and further processed using BoltzTraP2. The computed and experimental conductivity values are summarized in Table 2.

**Figure 2:**
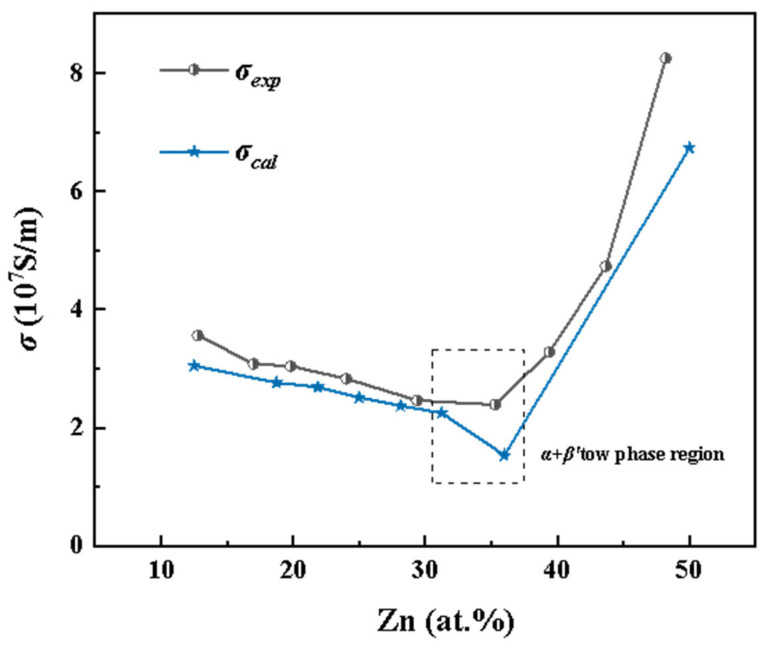
Curve of Cu–Zn alloy conductivity with Zn content, where the blue star curve (*σ_cal_*) is the calculated results, and the black dot curve (*σ_exp_*) is the experimental results [8].

Figure 2 compares the experimental and calculated electrical conductivities of Cu–Zn alloys as a function of Zn concentration. Overall, both data sets exhibit excellent agreement in terms of trends and conductivity variations across different phase regions, validating the effectiveness of the first-principles approach. In the α-phase region (FCC random solid solution, Zn < 35 at.%), both experimental and theoretical results show a decreasing conductivity with increasing Zn content. This behavior is attributed to the increased lattice distortion caused by the random substitution of Zn atoms, which enhances electron scattering, shortens the mean free path of conduction electrons, and consequently reduces electrical conductivity.

In the α + β′ two-phase region (Zn = 35–50 at.%), the β′ phase (ordered BCC structure) begins to form and increases in proportion with Zn content. Experimentally, as the alloy transitions from the α single-phase to the α + β′ two-phase region, the conductivity trend levels off, followed by a noticeable increase when Zn exceeds 37.04 at.%. However, a slight drop in the calculated conductivity at 36 at.% Zn is observed. This discrepancy is likely due to the simplifications in the two-phase structural model, which cannot fully capture the complex interfacial features present in real materials, thereby affecting the accuracy of the calculated conductivity.

At approximately 50 at.% Zn, the alloy enters the β′ single-phase region, where the experimental conductivity reaches 8.25 × 10^7^ S/m, significantly higher than that of the α-phase region. The calculated results show excellent agreement with this experimental trend. The enhanced conductivity in the β′ phase is attributed to its ordered BCC structure, which reduces electron scattering and improves the continuity of electron transport by minimizing lattice imperfections.

In summary, the calculated electrical conductivities of Cu–Zn alloys using the first-principles calculations show good agreement with experimental data across different phases and compositions. This confirms the feasibility of using first-principles approaches to predict electrical conductivity variations in metal alloys.

### 3.3. Effect of Zn Content on the Electronic Structure

To gain deeper insight into the observed decrease in electrical conductivity of the α-phase Cu–Zn alloy with increasing Zn content, we analyzed the electronic structure by calculating the density of states (DOS) at various Zn concentrations. The DOS describes the number of available electronic states per unit energy interval. In a given metal phase, small compositional changes can lead to variations in the DOS near the Fermi level. In Cu–Zn alloys, the gradual incorporation of Zn alters the electronic state distribution, which in turn affects electron transport properties. This enables us to quantify the influence of Zn content on conductivity from an electronic structure perspective.

**Figure 3:**
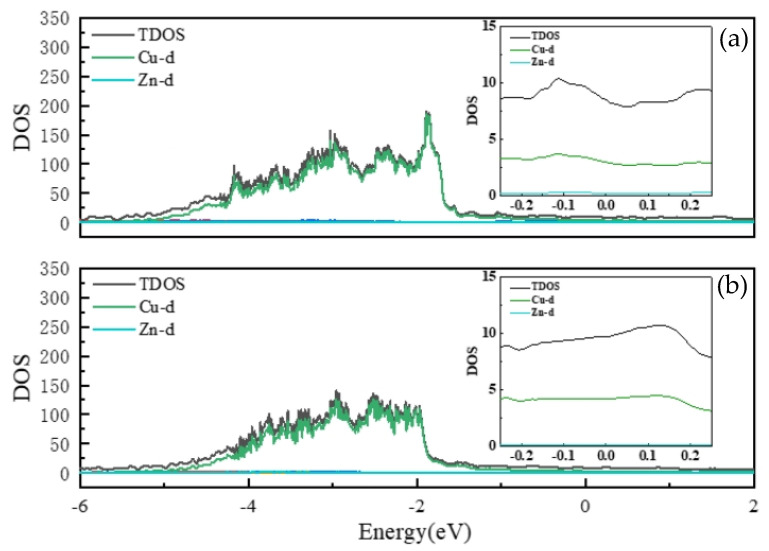
Total state density (TDOS) of the α phase of Cu–Zn alloy and partial wave state density (PDOS) of d-orbitals of Cu and Zn elements. (**a**) 12.5% Zn content; (**b**) 31.25% Zn content. The small graph on the right is a local magnification of the total state density near the Fermi surface.

Figure 3 presents the total density of states (TDOS) and the projected density of states (PDOS) of Cu and Zn d-orbitals for α-phase Cu–Zn alloys at 12.5 at.% and 31.25 at.% Zn. The contributions from s- and p-orbitals are comparatively small; a detailed discussion of their characteristics is provided in Figure S1 of the Supplemental Materials.

Additionally, since the Zn d-orbitals are mainly localized between –8 eV and –7 eV, no prominent Zn d peaks appear in the energy range of interest. As observed in Figure 3, the DOS near the Fermi level is dominated by Cu d-orbitals, which serve as the primary contributors to electrical conduction.

The TDOS values near the Fermi level for six α-phase structures are listed in Table 3. These values are obtained by integrating the TDOS in the energy window from –0.25 eV to 0.25 eV. The results indicate a clear decreasing trend in the DOS at the Fermi level with increasing Zn content, which aligns with the observed decline in electrical conductivity. This trend can be attributed to the reduced number of available electronic states near the Fermi level upon Zn addition, thereby hindering electron transport and decreasing conductivity. Although Zn s- and p-orbitals provide some contribution near the Fermi level, their DOS is significantly lower than that of Cu d-orbitals, resulting in a negligible impact on the overall conductivity.

### 3.4. Thermal Conductivity: Results and Discussion

Aoyama, S., and Ito, T., experimentally measured the thermal conductivity of Cu–Zn alloys in 1940 [8], and their data are used in this study as a reference standard for evaluating the calculated results. We investigated the variation in thermal conductivity of Cu–Zn alloys with Zn content and phase transitions between the α and β′ phases. Two different methods were employed to calculate the thermal conductivity at 78 K: the Wiedemann–Franz law and a first-principles approach combining density functional theory (DFT) with the Boltzmann transport theory. The corresponding results are summarized in Table 4. In the table, *σ* represents the experimentally measured electrical conductivity at 78 K, ***κ_exp_*** denotes the experimental thermal conductivity, ***κ_WF_*** is the thermal conductivity derived using the Wiedemann–Franz law, and ***κ_DFT_*** refers to the result obtained from DFT-based calculations.

**Figure 4:**
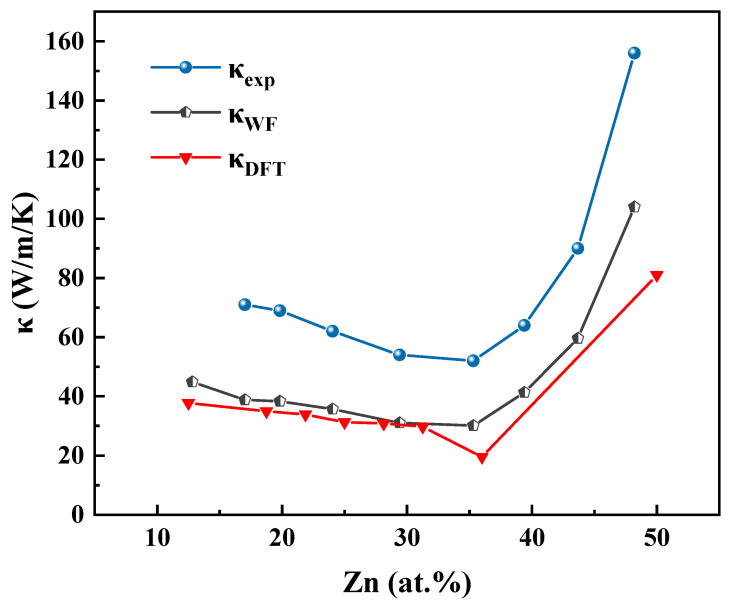
The thermal conductivity curves of the Cu–Zn binary alloy with different Zn contents at 78 K of the experimental data (***κ_exp_***), Wiedemann–Franz Law calculation results (***κ_WF_***), and DFT calculation results (***κ_DFT_***).

Figure 4 illustrates the thermal conductivity of Cu–Zn alloys at 78 K, based on the data in Table 4. The traditional method for calculating lattice (phonon) thermal conductivity involves solving the phonon Boltzmann transport equation using third-order interatomic force constants [44,45]. However, determining these third-order force constants becomes increasingly complex and computationally demanding as the number of atoms in the primitive cell increases. Moreover, the lattice contribution to the total thermal conductivity in Cu–Zn alloys has been reported to be relatively minor, typically accounting for only 4–7% of the total thermal conductivity [31]. Therefore, it is reasonable to neglect the phonon contribution in this study.

The experimental thermal conductivity values (***κ_exp_***) are slightly higher than those predicted by the Wiedemann–Franz law (***κ_WF_***), yet they are very close in magnitude. This suggests that although the Wiedemann–Franz approach omits the phonon contribution, it still provides a reasonably accurate estimate of the total thermal conductivity in Cu–Zn alloys. This further confirms that the electronic component dominates the thermal transport in these alloys.

Comparison between the experimental data (***κ_exp_***) and the DFT-calculated results (***κ_DFT_***) shows that ***κ_exp_*** is slightly higher than ***κ_DFT_***, but the values are close and follow a consistent trend. This agreement among ***κ_exp_***, ***κ_WF_***, and ***κ_DFT_*** supports the conclusion that electron thermal conductivity is the dominant contributor to the total thermal conductivity in Cu–Zn alloys and also validates the accuracy and reliability of the thermal conductivity calculations presented in this study.

By comparing the electrical conductivity and thermal conductivity curves of Cu–Zn alloys in Figure 2 and Figure 4, a clear positive correlation between the two properties with respect to Zn atomic concentration can be observed. When the Zn content is below 35 at.%, both electrical and thermal conductivities decrease with increasing Zn concentration, reaching their minimum at approximately 35 at.% Zn. Beyond this point, both properties exhibit a rapid increase as Zn content continues to rise. The two sets of curves demonstrate a highly synchronized trend. This behavior reflects the dominant role of electronic transport characteristics in determining both the electrical and thermal conductivities of Cu–Zn alloys. In summary, the accuracy of electrical conductivity calculations directly influences the reliability of thermal conductivity predictions. Therefore, improving the precision of electrical conductivity calculations is essential for enhancing the overall accuracy of thermal conductivity modeling in computational studies.

## 4. Conclusions

In this study, eight crystal structure models of Cu–Zn binary alloys were constructed, covering three types of phase configurations within the Zn concentration range of 0–50 at.%: α single phase (FCC), α + β′ dual phase, and β′ single phase (BCC). The electrical and thermal conductivities of these models were calculated using the first-principles calculations in combination with the Boltzmann transport equation, and the electronic structure of the α-phase configurations was further investigated. The results show that both electrical and thermal conductivities of Cu–Zn alloys exhibit a decreasing–increasing trend with increasing Zn content from 0 to 50 at.%, displaying a clear positive correlation between the two properties. This trend is in good agreement with experimental observations. Electronic structure analysis further reveals that, in the α-phase region, the density of states (DOS) near the Fermi level is mainly contributed by the Cu d-orbitals. As Zn content increases, the effective DOS near the Fermi level decreases, leading to a reduction in electron transport capability and, consequently, a decline in electrical conductivity within the α-phase region. Regarding thermal conductivity calculations, both the Wiedemann–Franz law and first-principles methods were employed, and the calculated results exhibit trends consistent with experimental data. In summary, this work systematically investigates the variation of electrical and thermal transport properties of Cu–Zn binary alloys with Zn content and elucidates the underlying physical mechanisms from the perspective of electronic structure. The findings provide theoretical insights to support transport property studies and performance optimization in complex alloy systems.

## Figures and Tables

**Table 1:** Calculated lattice parameters and experimental crystallographic data of pure copper, pure zinc, and Cu–Zn intermetallic compounds.

Phase	Crystal Structure	Lattice Parameters (Å)		Ref.
Cu	FCC	*a* = 3.6077		Exp. [40]
		*a* = 3.6072		Exp. [41]
		*a* = 3.615		This study
Cu_3_Zn-*α*	FCC	*a* = 3.6718		Exp. [43]
		*a* = 3.677		This study
CuZn-β’	BCC	*a* = 2.9575		Exp. [44]
		*a* = 2.921		This study
Zn	HCP	*a* = 2.6594	*c* = 4.9368	Exp. [42]
		*a* = 2.6190	*c* = 4.986	This study

**Table 2:** Experimental [8] and calculated conductivity of Cu–Zn binary alloy.

Zn at.%	12.81	17.02	19.81	24.04	29.41	35.3	39.39	43.68	48.02
Conductivity (experimental) 10^7^ S/m	3.56	3.08	3.04	2.83	2.46	2.39	3.28	4.73	8.25
Zn at.%	12.5	18.75	21.875	25	28.125	31.25	36	-	50
Conductivity (computed) 10^7^ S/m	2.02	1.83	1.78	1.66	1.57	1.49	1.02	-	4.46

**Table 3:** TDOS integrals of six *α* phases of Cu–Zn alloy between −0.25 eV and 0.25 eV near the Fermi surface.

Zn Doping Ratio	12.5 at.%	18.75 at.%	21.875 at.%	25 at.%	28.125 at.%	31.25 at.%
Integrated TDOS Value	1.96	1.93	1.89	1.85	1.83	1.78

**Table 4:** Thermal conductivity of Cu–Zn binary alloy at 78 K.

**Results of Wiedemann–Franz Law Calculation Method**								
Zn at.%	17.01	19.92	24.04	29.41	35.30	39.39	43.68	48.20
***κ_exp_*** (W/m/K)	71	69	62	54	52	64	90	156
***κ_WF_*** (W/m/K)	38.82	38.31	35.67	31.01	30.12	41.34	59.62	103.98
**Results of DFT calculation method**								
Zn at.%	12.5	18.75	21.875	25	28.125	31.25	36	50
***κ_DFT_*** (W/m/K)	37.74	34.97	33.92	31.26	30.83	29.73	19.55	80.99

## Data Availability

The original contributions presented in this study are included in the article/Supplementary Materials. Further inquiries can be directed to the corresponding authors.

## Supplementary Materials

The following supporting information can be downloaded at: https://www.mdpi.com/article/10.3390/ma18102310/s1, Figure S1: Calculated PDOS of Zn-s and Zn-p in the structures of (a) β′ single phase and (b) α single phase(Zn 12.5% content).
