# Supplementary material for: First-Principles Study on the Electrical and Thermal Conductivities of Cu–Zn Binary Alloys
Source: Materials (Basel). 2025 May 15;18(10):2310. doi: 10.3390/ma18102310 (PMC12113239; doi:10.3390/ma18102310)
Supplement: Supplementary file 1 [file materials-18-02310-s001.zip › materials-3602726-supplementary.pdf]

Supplemental Materials for

"First-Principles Study on the Electrical and Thermal Conductivities of Cu-Zn Binary Alloys"

HUANG Lei [1], PENG Bo [2], YU Qinchi [2], HUANG Guojie [1,*], WANG Changhao [2,*], WANG Ruzhi [2], TIAN Ning [1]

[1] China Nonferrous Metals Innovation Institute (Tianjin) Co., Ltd., High-Performance Copper Alloy Materials Research Institute, Tianjin 300393, China; huanglei.my@foxmail.com (H.L.)

[2] State Key Laboratory of Materials Low-Carbon Recycling, College of Material Science and Engineering, Beijing University of Technology, Beijing 100124, China;

Ⅰ. PDOS of Zn s/p-orbital

In order to elucidate Zn s/p-orbital effects, we conducted a dedicated analysis of their contributions.

The Zn-s orbitals predominantly form deep-level localized states, which exhibit negligible influence on electron transport near the Fermi level and the behavior of the valence band maximum (VBM). Their primary contribution lies in chemical bonding energy. Meanwhile, Zn-p orbitals exhibit broader energy distribution with enhanced band delocalization, indicating their participation in shallow-level bonding and electron migration. However, compared to the Cu-d orbitals, their contribution to electron mobility remains substantially weaker.

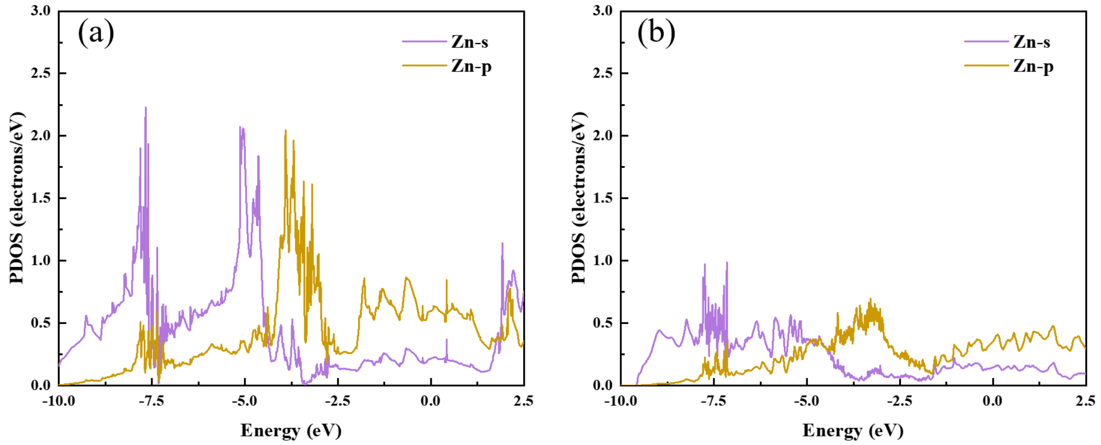

Figure S1. Calculated PDOS of Zn-s and Zn-p in the structures of (a) β′ single phase and (b) α single phase(Zn 12.5% content)
